# Stress-Induced Hyperprolactinemia Mimicking Pituitary Pathology: A Diagnostic Challenge

**DOI:** 10.7759/cureus.85821

**Published:** 2025-06-12

**Authors:** Kanika Vats, Roshan Kurian John, Sindhu Ann Korah

**Affiliations:** 1 Directorate of Research and Innovation, Emirates Classification Society (TASNEEF), Abu Dhabi, ARE; 2 Graduate School of Biomedical Studies, Icahn School of Medicine at Mount Sinai, New York, USA; 3 Department of Obstetrics and Gynecology, New Medical Centre (NMC) Specialty Hospital, Abu Dhabi, ARE

**Keywords:** dienogest, endometriosis, hyperprolactinemia, interdisciplinary communication, neuroendocrinology, neuro-ophthalmology, pituitary adenoma, prolactin, psychological stress, vision disorders

## Abstract

Hyperprolactinemia is often linked to pituitary adenomas, medications, or systemic disorders, but it can also be triggered by stress. The relationship between stress and elevated prolactin levels is complex and requires thorough evaluation to rule out underlying pathological causes.

We present the case of a 37-year-old non-pregnant female with a known history of ovarian endometriosis, diagnosed more than a decade ago and currently managed with dienogest 2 mg daily. During a routine gynecological follow-up, laboratory investigations revealed mildly elevated serum prolactin levels. The patient concurrently reported the recent onset of acne, intermittent blurry vision, and occipital headaches, symptoms that were not present in previous assessments.

Due to these atypical features, a multidisciplinary evaluation was initiated to explore possible underlying causes, including pituitary enlargement. Pelvic ultrasonography confirmed stable findings with no new endometriotic lesions. MRI of the brain showed no evidence of pituitary adenoma or structural abnormalities. Comprehensive ophthalmological examination demonstrated normal macular health in both eyes; however, visual field testing (perimetry) detected a central scotoma in the right eye.

With no identifiable secondary causes and a recent history of psychological stress, a diagnosis of stress-induced hyperprolactinemia was considered. This case underscores the diagnostic complexity of endocrine abnormalities when neuro-ophthalmologic symptoms are present without radiologic findings. It highlights the importance of considering psychological stress as a potential contributor and reinforces the need for a holistic, multidisciplinary approach to avoid overdiagnosis and ensure appropriate management.

## Introduction

Prolactin is a peptide composed of 198 amino acids and shares genetic, structural, and receptor-binding characteristics with growth hormone and human placental lactogen [1]. Lactotrophs, which make up approximately 20% of the cells in the anterior pituitary, primarily in its lateral region, are responsible for prolactin secretion [2]. Prolactin regulation is predominantly inhibitory, with dopamine acting through pituitary dopamine type 2 receptors to suppress its release [1].

Hyperprolactinemia, defined by elevated serum prolactin levels, arises from various etiologies, including pituitary adenomas, medication adverse effects, and systemic disorders [3]. Notably, stress has been identified as a significant yet often overlooked contributor to increased prolactin secretion [4]. The physiological mechanisms underlying stress-induced hyperprolactinemia involve the activation of the hypothalamic-pituitary-adrenal (HPA) axis, leading to increased prolactin release [5]. This response is considered an adaptive mechanism to counteract stress, but chronic activation may result in sustained hyperprolactinemia with various clinical manifestations, including reproductive dysfunction and neurological symptoms [6].

Endometriosis, a chronic gynecological condition characterized by the presence of endometrial tissue outside the uterine cavity, has also been associated with elevated prolactin levels [7]. Studies have demonstrated higher prolactin concentrations in patients with endometriosis compared to controls, suggesting a possible role of prolactin in disease progression and infertility [8]. The interaction between stress, prolactin, and endometriosis remains a topic of ongoing research, as stress can further disrupt hormonal balance and exacerbate symptoms [9].

Studies have also demonstrated a correlation between peak prolactin and luteinizing hormone (LH) secretion in women with polycystic ovary syndrome (PCOS) [10]. It is recommended to investigate elevated prolactin levels in PCOS patients to identify the underlying causes of hyperprolactinemia, particularly macroprolactinemia [11].

This case report presents a hyperprolactinemia case in a patient with an established history of ovarian endometriosis. The patient’s clinical presentation, diagnostic workup, and the role of stress in contributing to elevated prolactin levels are discussed.

## Case presentation

Patient information

A 37-year-old non-pregnant Asian woman attended a routine gynecological follow-up at an outpatient clinic in a general hospital located in the Emirate of Abu Dhabi, United Arab Emirates. She had a known history of ovarian endometriosis diagnosed 10 years prior, managed with laparoscopic adhesiolysis at the time. Since then, she has been stable on long-term dienogest 2 mg daily. She had no reported comorbidities, no relevant family history, and no recent changes in medication apart from the continued use of dienogest.

Chief complaints and history of present illness

Although the visit was routine, the patient reported new-onset symptoms, including intermittent blurred vision over the past 15 days, persistent occipital headaches for four months, and mild acne. The episodes of blurred vision were transient, occurring a few times a week, and the headaches were dull, occasionally throbbing in nature. These symptoms were new and had not been previously documented.

Menstrual and medication history

Her menstrual cycles remained irregular, consistent with the expected amenorrheic effect of continuous dienogest use. She denied experiencing galactorrhea or breast tenderness but reported gradual weight gain over the past two years. She remained compliant with her dienogest regimen and was not taking any other medications.

Physical examination

Vital signs were within normal limits: blood pressure 121/84 mmHg, heart rate 78 bpm, and temperature 36.8°C. Her BMI was 28.3 kg/m² (mildly overweight). General physical and systemic examinations were unremarkable. Mild acneiform lesions on the cheeks, chin, and forehead were evident.

Laboratory investigations

Given her presenting symptoms, the gynecology clinic performed a set of routine fasting laboratory tests as part of the initial baseline assessment to exclude possible infection, anemia, or other hematologic disorders that might explain her nonspecific systemic symptoms. This workup included a hematology panel with a complete blood count (CBC) and differential, as detailed in Table 1. All values in the CBC were within normal limits.

**Table 1:** Initial CBC with differential (EDTA) in gynecological assessment CBC: complete blood count, EDTA: ethylenediaminetetraacetic acid, fL: femtoliters, gm/dL: grams per deciliter, L: liter, MCV: mean corpuscular volume, MCH: mean corpuscular hemoglobin, pg: picograms, PCV: packed cell volume, RBC: red blood count, WBC: white blood count

Test description	Result	Reference range
Haemoglobin	13.0 gm/dL	12-15 gm/dL
WBC count, total	9.2 x 10^9/L	4-10 x 10^9/L
RBC count	4.5 x 10^12/L	3.8-4.8 x 10^12/L
Platelets	264 x 10^9/L	150-410 x 10^9/L
Hematocrit (PCV)	40%	36-46%
MCV	90 fL	83-101 fL
MCH	29 pg	27-32 pg
MCH concentration	32 gm/dL	32-35 gm/dL
Red cell distribution width	13.1%	11.6-14.0%
Neutrophils, differential	56.0%	40-80%
Lymphocytes, differential	36.8%	20-40%
Monocytes, differential	5.7%	2-10%
Eosinophils, differential	1.1%	1-6%
Basophils, differential	0.4%	<2%
Absolute neutrophil count	5.2 x 10^9/L	2-7 x 10^9/L
Absolute lymphocyte count	3.4 x 10^9/L	1-3 x 10^9/L
Absolute monocyte count	0.5 x 10^9/L	0.2-1.0 x 10^9/L
Absolute eosinophil count	0.1 x 10^9/L	0.02-0.5 x 10^9/L
Absolute basophil count	0.0 x 10^9/L	0.02-0.1 x 10^9/L

Additionally, considering the presence of acne and a history of endometriosis, PCOS was included in the differential diagnosis. As a result, hormonal imbalances commonly associated with PCOS were assessed. The biochemistry workup included fasting glucose, lipid profile, thyroid-stimulating hormone (TSH), follicle-stimulating hormone (FSH), lactate dehydrogenase (LDH), insulin, and serum prolactin, with the results outlined in Table 2. All values were within normal limits, except for a slightly elevated serum prolactin level of 609.10 µIU/mL.

**Table 2:** Initial biochemistry panel in gynecological assessment CHD: coronary heart disease, FSH: follicle-stimulating hormone, HDL: high-density lipoprotein, L: liter, LDL: low-density lipoproteins, LDH: lactate dehydrogenase, mIU/L: one-thousandth of an international unit, mU/L: milliunits per liter, mmol/L: millimoles per liter, TSH: thyroid-stimulating hormone

Test description	Result	Reference range
Cholesterol HDL (serum)	0.96 mmol/L	<1.03 mmol/L - major risk for CHD
		≥1.55 mmol/L - negative risk for CHD
TSH (serum)	2.05 mIU/L	0.2-4.2 mIU/L
Glucose, fasting plasma	5.03 mmol/L	Diabetes: ≥7.0 mmol/L
		Prediabetes: 5.6-6.9 mmol/L
		Normal: <5.6 mmol/L
Triglycerides (serum)	1.32 mmol/L	Normal: <1.69 mmol/L
		High: 1.69-2.25 mmo/L
		Hypertriglyceridemic: 2.26-5.64 mmol/L
		Very high: >5.64 mmol/L
Cholesterol total (serum)	4.47 mmol/L	Desirable: <5.18 mmol/L
		Borderline high: 5.18-6.19 mmol/L
		High: >6.19 mmol/L
Cholesterol LDL (serum)	3.10 mmol/L	Optimal: <2.59 mmol/L
		Near optimal: 2.59-3.34 mmol/L
		Borderline high: 3.37-4.12 mmol/L
		High: 4.14-4.89 mmol/L
		Very high: ≥4.92 mmol/L
FSH (serum)	5.25 mIU/ml	Follicular phase: 3.5-12.5 mIU/ml (women)
		Ovulation phase: 4.7-21.5 mIU/ml (women)
		Luteal phase: 1.7-7.7 mIU/ml (women)
		Postmenopasue: 25.8-134.8 mIU/ml (women)
LDH (serum)	149.00 U/L	135-214 U/L (women)
Prolactin (serum)	609.10 uIU/ml	102-496 uIU/ml (women)
Insulin-fasting, serum	10.14 mU/L	3.0-25

Clinical evaluation and diagnostic workup

Following the finding of mildly elevated serum prolactin levels (609.10 uIU/mL), and in the absence of a history of medications known to elevate prolactin (such as antipsychotics or antidepressants), the patient was referred for pelvic ultrasonography to assess for possible recurrence of endometriotic lesions or features suggestive of PCOS. This was done despite FSH and LH levels appearing within the normal range, which typically would not explain the rise in prolactin.

Following the lab and ultrasonography reports, she reported persistent occipital headaches and intermittent blurred vision. Due to the clinical significance of these symptoms, further diagnostic workup was initiated, leading to a referral to the neurology clinic for assessment of potential pituitary involvement.

During the neurological evaluation, a psychosocial assessment identified significant physiological stress over the past six months. The patient described dull, occasionally throbbing occipital headaches that had been ongoing for four months and transient blurred vision over the preceding 15 days. However, there were no clinical signs of anxiety or depression.

These neurological symptoms, coupled with the mildly elevated serum prolactin level of 609.10 µIU/mL, raised concerns for potential intracranial pathology, particularly pituitary involvement, such as a pituitary adenoma. Prolactin-secreting tumors, or prolactinomas, can lead to elevated prolactin levels and cause symptoms like headaches and visual disturbances due to compression of nearby structures, like the optic chiasm. This raised concern and triggered the need for a non-contrast brain MRI to investigate the central causes of her symptoms. Prior to the scan, serum creatinine was measured and found to be within normal limits (74 μmol/L).

She was prescribed methylcobalamin 500 mcg once daily for one month to support neural health and paracetamol 500 mg as needed for headache relief. She was advised to return for a follow-up visit as soon as the MRI reports were available.

Simultaneously, an ophthalmology referral was initiated from the neurology clinic to evaluate potential optic pathway involvement, given the patient's complaints of transient blurred vision and the concern for possible pituitary tumor-related compression of the optic chiasm. To further investigate this, diagnostic tests were ordered, including optical coherence tomography (OCT) to assess macular structure and detect any abnormalities in the retina or optic nerve. Additionally, visual field testing (perimetry) was performed to evaluate the functional impact on the patient's vision and detect any deficits that could indicate optic nerve or chiasmal involvement.

Imaging findings

**Figure 1:**
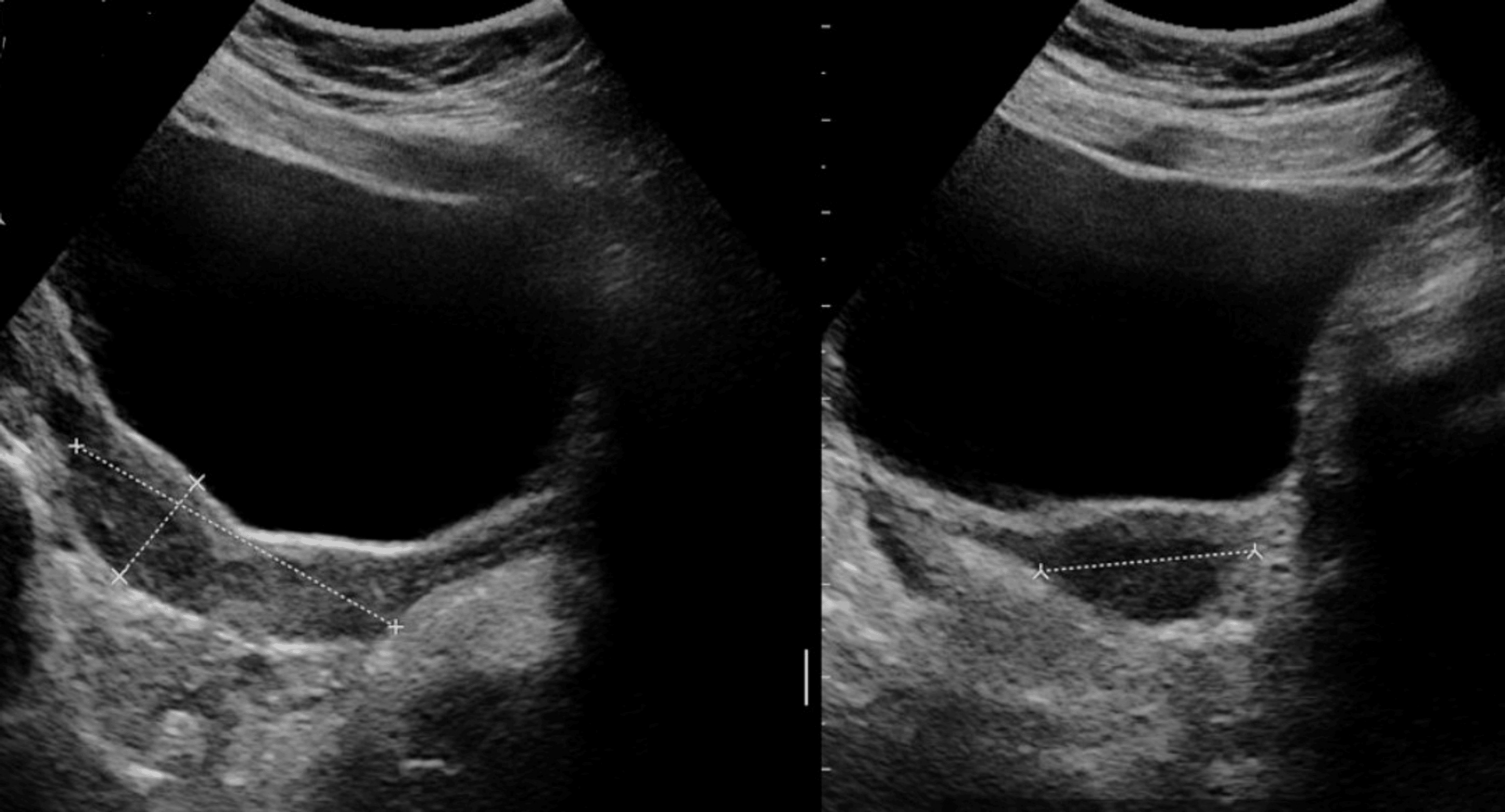
Pelvic ultrasonography showing normal morphology (uterus)

Both ovaries were normal in size and echotexture, with no adnexal masses or free pelvic fluid (Figure 1). The uterus measured 80 × 30 mm with preserved contour and no focal lesions. Endometrial thickness and cervix appeared normal.

**Figure 2:**
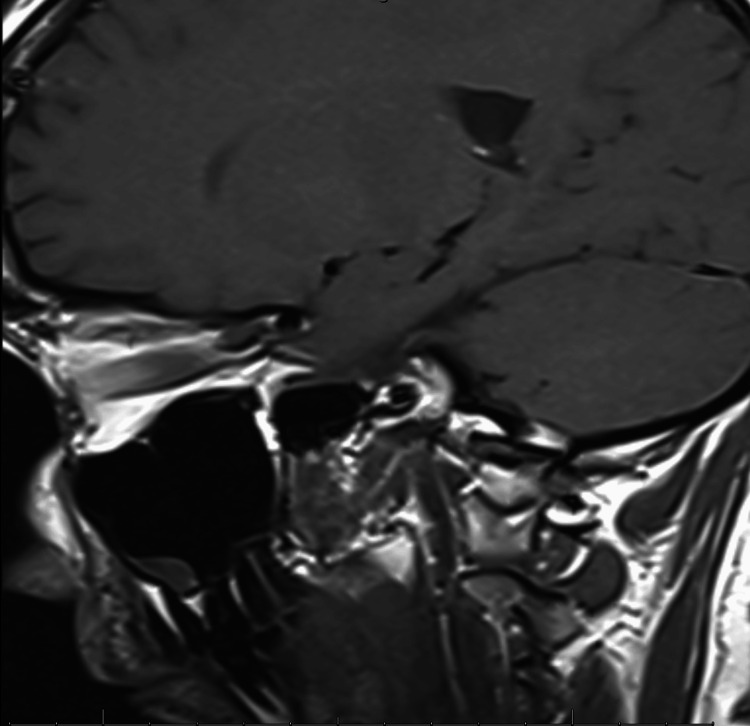
Sagittal view of brain MRI showing normal pituitary gland morphology MRI: magnetic resonance imaging

**Figure 3:**
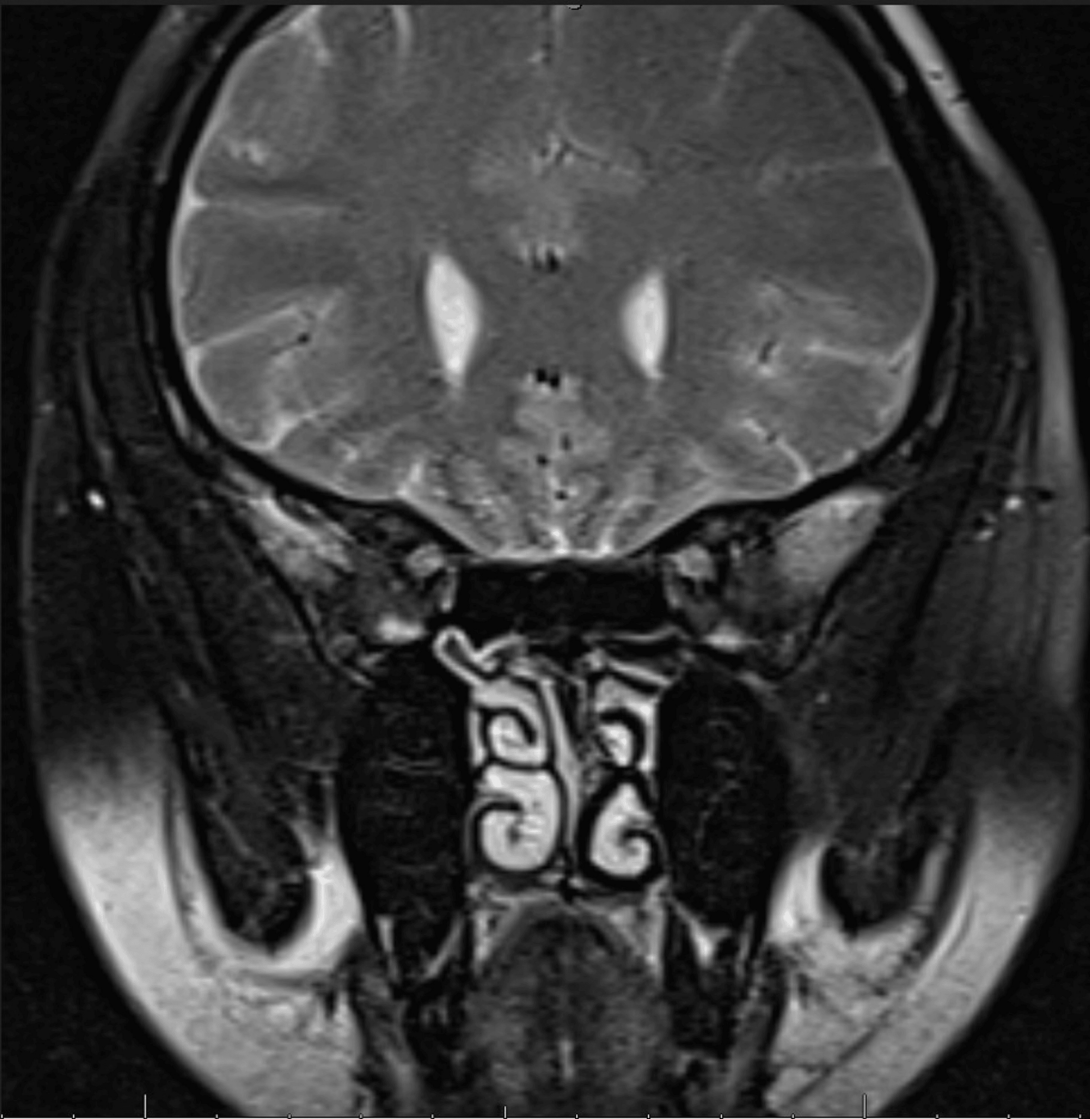
Coronal view of brain MRI showing normal pituitary gland morphology MRI: magnetic resonance imaging

Conducted using a 1.5T MRI scanner (Siemens Healthcare Inc., Erlangen, Germany), the scan showed a pituitary gland of normal size and morphology with preserved posterior lobe signal (Figures 2-3). No adenomas, hemorrhage, or structural abnormalities were observed. No significant intracranial pathology was identified.

**Figure 4:**
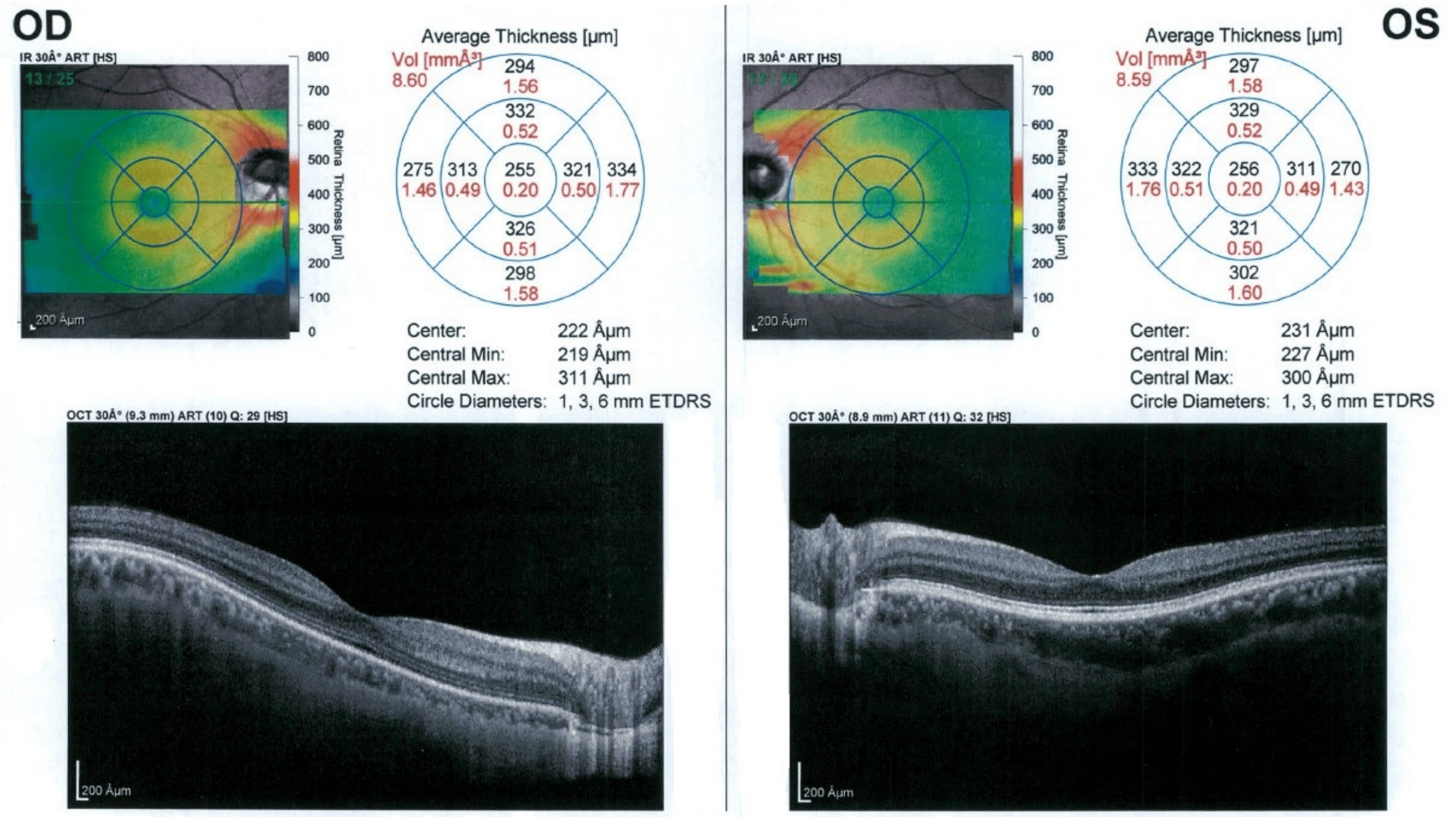
OCT imaging with intact macular structure in both eyes ETDRS: Early Treatment Diabetic Retinopathy Study, OD: right eye, OCT: optical coherence tomography, OS: left eye

OCT indicates a normal macular structure in both eyes (Figure 4).

**Figure 5:**
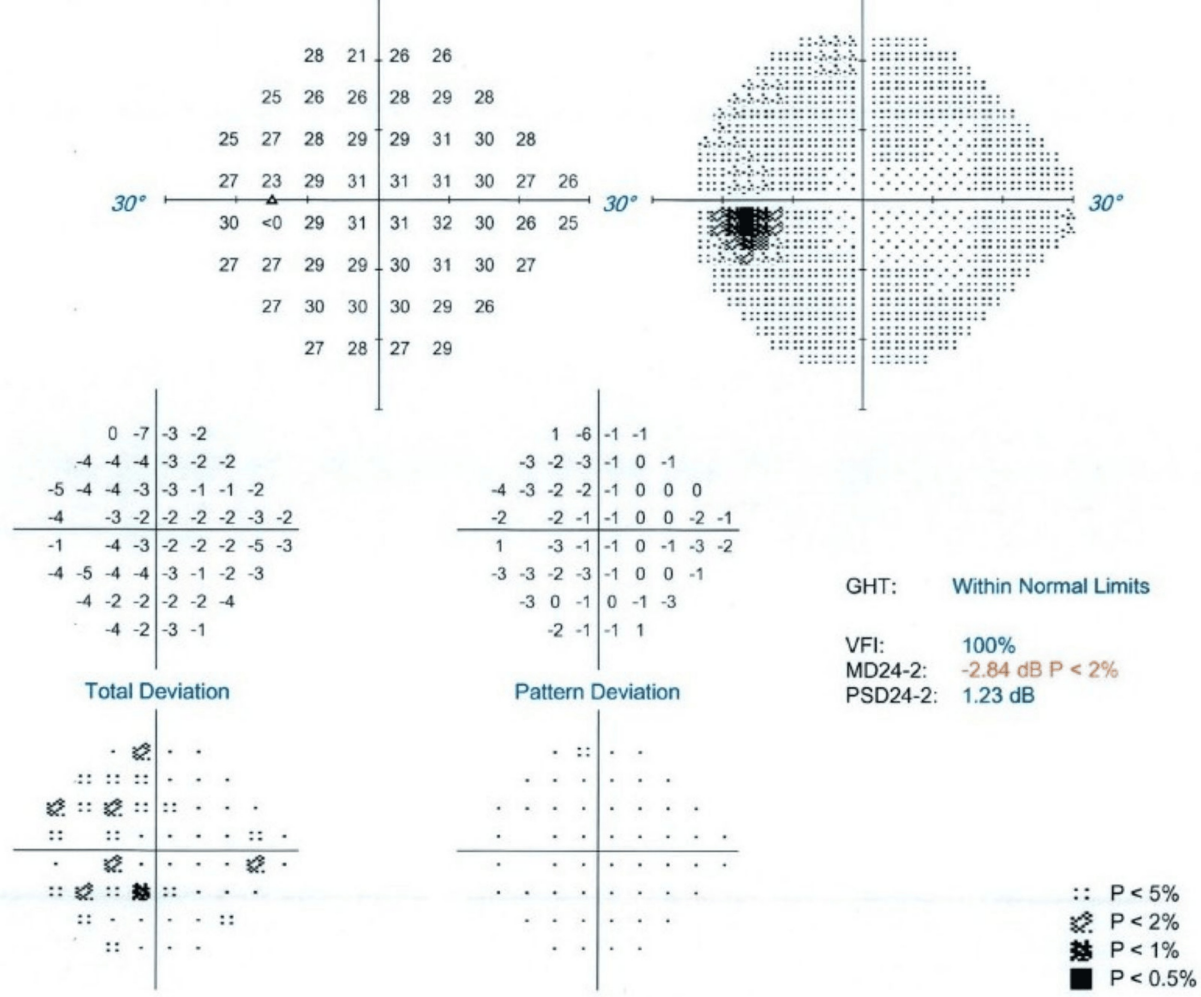
Visual field test (perimetry) images of the left eye GHT: glaucoma hemifield test, MD: median deviation, PSD: pattern standard deviation, VFI: visual field index

**Figure 6:**
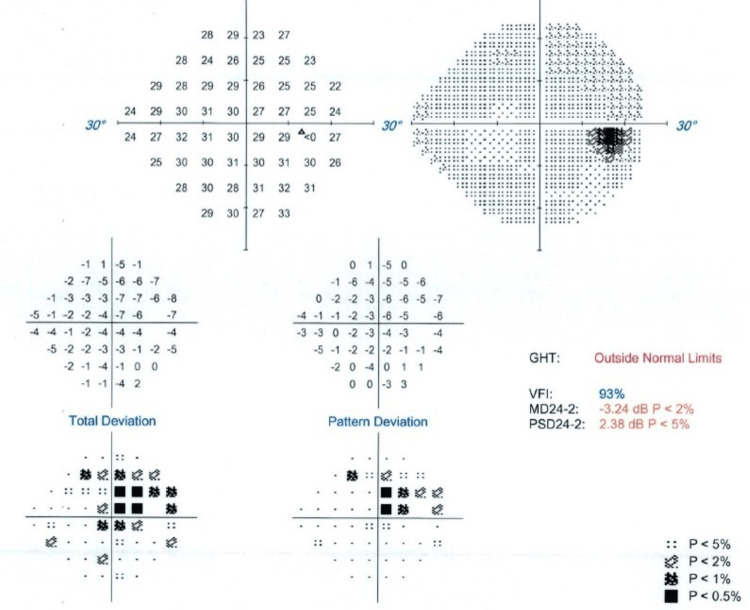
visual field test (perimetry) images of the right eye GHT: glaucoma hemifield test, MD: median deviation, PSD: pattern standard deviation, VFI: visual field index

The left eye showed results within the normal range, with a visual field index (VFI) of 100%. In contrast, the right eye had a VFI of 93%, suggesting the presence of a central scotoma (Figures 5-6).

Follow-up care and outcome

The patient was scheduled for follow-up appointments in the gynecology, neurology, and ophthalmology clinics to review and coordinate care based on the results of all diagnostic investigations. The gynecology clinic cleared the patient of any recurrence of endometriotic lesions or features suggestive of PCOS.

Given the normal brain MRI findings with no abnormalities detected, the neurology clinic found no evidence suggestive of a pituitary adenoma. Her headaches, along with the episodes of blurred vision, were believed to be related to physical stress, and she was advised to monitor her symptoms over the following week.

From the ophthalmology clinic, although visual acuity was preserved on OCT, a central scotoma noted on visual field testing was considered subjective and likely stress-related, possibly due to the emotional strain of repeated hospital visits, tests, and appointments. Since no abnormalities were found on the brain MRI, she was advised to keep an eye on her symptoms and repeat the visual field test in six weeks if they continued.

At the two-week follow-up with her primary gynecologist, the patient reported complete resolution of her symptoms, including headaches and blurred vision, and confirmed that her stress levels had recently decreased. She declined further investigation of the mildly elevated prolactin level to check if it had returned to normal.

It was concluded that the elevated prolactin and associated symptoms were most likely stress-induced, in line with her improved well-being and reduced stress levels.

## Discussion

Hyperprolactinemia is most commonly associated with pituitary adenomas (particularly prolactinomas), hypothyroidism, pregnancy, lactation, and certain medications such as antipsychotics and antidepressants. However, when no physical cause is found, functional factors, especially psychological stress, are increasingly recognized as possible reasons for elevated serum prolactin levels [12].

In this case, the patient exhibited mildly elevated prolactin levels alongside symptoms such as acne, blurred vision, and occipital headaches. These clinical features warranted a comprehensive evaluation to assess the underlying cause. MRI revealed no pituitary abnormalities, and the patient had no history of medications known to induce hyperprolactinemia.

Interestingly, the ophthalmological examination revealed a central scotoma in the right eye, raising initial concern for optic pathway involvement. However, the absence of radiologic abnormalities and normal macular findings on OCT reduced the likelihood of an organic lesion. Moreover, as visual field testing (perimetry) is a subjective assessment that relies heavily on the patient's attention and cooperation, false positives can occur, particularly in individuals under psychological stress [13]. Therefore, a functional cause was considered more likely.

Chronic use of dienogest, a synthetic progestin commonly prescribed for the management of endometriosis, has not been strongly associated with hyperprolactinemia. On the contrary, some studies suggest that dienogest may reduce serum prolactin levels [14-16]. Additionally, due to its antiandrogenic properties, acting as an androgen receptor antagonist, and its favorable metabolic profile, dienogest is also considered a potential therapeutic option for the management of PCOS [17]. These factors further support the exclusion of a pharmacologic cause for hyperprolactinemia in this patient.

It has been suggested that accelerated gonadotropin-releasing hormone (GnRH) pulsatility in women with PCOS may contribute to elevated LH levels and a reduction in dopaminergic tone, potentially leading to hyperprolactinemia. However, studies have shown no significant decrease in prolactin levels among PCOS patients undergoing pituitary desensitization with GnRH agonists [10]. Moreover, in this case, the patient's LH levels were within the normal range, making this mechanism less likely.

Psychological and physiological stress is known to stimulate HPA axis activity, resulting in elevated cortisol and, indirectly, prolactin levels. Acute and chronic stress have been associated with transient prolactin elevations through serotonergic and dopaminergic modulation [18,19]. In women, particularly, stress-induced hyperprolactinemia has been connected to menstrual irregularities, galactorrhea, and even infertility [20].

A multidisciplinary approach, incorporating gynecology, neurology, and ophthalmology, was critical in reaching a non-invasive, patient-centered conclusion.

## Conclusions

This case illustrates the intricate relationship between elevated prolactin levels, neurological symptoms, and psychological stress. Although there were initial concerns about potential pituitary abnormalities and optic pathway involvement, thorough diagnostic investigations, including brain MRI, pelvic ultrasound, and detailed eye examinations, revealed no significant structural issues. The patient’s occipital headaches and blurred vision, along with mildly high prolactin levels, were ultimately attributed to stress. Her symptoms improved significantly with supportive care and stress reduction, highlighting the critical role of psychosocial factors in evaluating and managing unexplained clinical presentations. Continued follow-up confirmed her complete recovery, reinforcing the impact of stress management on overall health and symptom resolution.

Furthermore, future prospective studies may contribute to the development of more definitive guidelines for managing patients with endocrinological, ophthalmological, and neurological symptoms while emphasizing the importance of psychosocial factors in diagnosis and treatment.
